# Assessing Pediatric Pelvic Fracture Patterns: New Insights and Unique Fracture Characteristics

**DOI:** 10.1097/BPO.0000000000003107

**Published:** 2025-09-22

**Authors:** Anna H.M. Mennen, Robert Hemke, Frank W. Bloemers, Abby E. Geerlings, Mario Maas, Daphne van Embden

**Affiliations:** Departments of * Surgery; † Radiology & Nuclear Medicine, Amsterdam UMC, University of Amsterdam, Meibergdreef 9, Amsterdam, The Netherlands

**Keywords:** pediatric pelvic fractures, classification, pelvic ring fractures, ped-LC3

## Abstract

**Background:**

Pediatric pelvic ring fractures are different in biomechanics and anatomy compared with adults. Existing classification systems are insufficient in assessing the mechanical stability of these fractures due to a variety of reasons, leading to a potential underestimation of the injury severity, resulting in suboptimal treatment with the risk of long-term dysfunctions. This study aims to address this problem by comprehensively describing a cohort of patients with pediatric pelvic fractures, identifying specific pediatric pelvic fractures and patterns.

**Methods:**

A retrospective cohort selection of pediatric patients with a pelvic fracture treated in a major level 1 trauma center between 2001 and 2021 was conducted. Fracture patterns were classified using existing systems (Tile, Young and Burgess, Torode and Zieg), with additional parameters such as skeletal maturity considered. In addition, the incidence of specific pediatric fracture characteristics was reviewed.

**Results:**

The CT scans of 68 children were reviewed. The median age was 15.5 years, with a majority being female (53%). Traffic accidents were the primary mechanism of injury (65%). Most fractures were classified as Tile type B2 (53%) and Young & Burgess LC3 (32%). SI-joint avulsion fractures were frequently seen (n=14, 21%), predominantly in children with a mature pelvis (n=10, 71%). A significant proportion of fractures did not fit conventional classifications, with little difference between skeletally mature and immature patients (73% vs. 75%).

**Conclusions:**

A large portion of skeletally mature and immature patients cannot be classified according to the currently existing classifications, highlighting the need for a tailored pediatric classification system. APC2-like fracture patterns had a high incidence of SI-joint avulsion fractures, so purely ligamentous APC-fracture patterns are, in our experience, very rare in children. In addition, a previously undescribed fracture pattern (ped-LC3) was identified. Future research is necessary to grasp the full concept of skeletal maturation on the biomechanics and distribution of forces in the pediatric pelvis.

**Level of Evidence:**

Level III

Pediatric pelvic ring fractures are relatively rare compared with adult pelvic fractures, but are increasing in numbers over the past 20 years.^1^ They have high rates of concomitant injuries and mortality related to neurotrauma.^2^ Historically, most pediatric patients with a pelvic fracture were treated nonoperatively. However, more recent studies show that treating mechanically unstable pelvic ring fractures nonoperatively could lead to long-term complaints like leg length discrepancy, chronic pain, and decreased mobility.^3,4^ Adequately assessing the mechanical stability of the pelvic ring, and thus the necessity for surgical stabilization, is key in treating these children and adolescents.

Currently, the most commonly used classification systems to assess pediatric pelvic fractures are the Tile classification and Torode and Zieg classification.^5^ However, both of these classifications come with limitations; they are either designed for the classification of adult pelvises or to assess the likelihood of concomitant injuries and thus focus on survival instead of mechanical stability. As studies have shown, the pediatric pelvis is not a scaled-down version of the adult pelvis, but has its own unique anatomic and biomechanical characteristics.^6–8^ For example, the pediatric pelvis has a higher proportion of cartilage and thus greater elasticity, incomplete ossification centers, and strong but elastic ligaments.^9^ This allows the pediatric pelvis to absorb and distribute forces differently than the adult pelvis, which plays a role in the fracture patterns that are observed in pediatric patients. A recent literature review has elaborated on some of these rare pelvic fractures which are unable to be classified using the current classification systems.^9^ Not being able to classify fracture patterns potentially hinders the accurate assessment of the mechanical stability of the pelvic ring and thus the need for surgical stabilization. In addition, none of the classification systems takes skeletal maturity into consideration. Although evidence on this topic is slim, 2 recent studies suggest that skeletal maturity does influence pelvic fracture patterns in children and adolescents.^10,11^

Our study aims to achieve a comprehensive understanding of pelvic ring fractures in pediatric patients by providing detailed descriptions of the fracture patterns. Second, we evaluate the adequacy of the current classification systems in pediatric patients and identify specific characteristics that differentiate pediatric pelvic ring fracture patterns from those in the adult population.

## METHODS

### Study Design and Study Setting

This study retrospectively reviewed all cases of pediatric pelvic ring fractures presented to the Amsterdam University Medical Centres between January 1, 2001, and December 31, 2021. All patients were identified from the Dutch trauma registry and electronic patient records. The Dutch National Trauma Registry (DNTR) is a nationwide database that includes all trauma patients admitted to Dutch hospitals. Data collection is mandatory for all hospitals, and registry data are prospectively recorded by trained data managers based on hospital admission records, imaging, and discharge summaries. Inclusion in the DNTR requires that patients be admitted following trauma and/or registered through the emergency department with trauma-related injuries. Data quality is maintained through standardized data entry protocols and regular national audits. For this study, only patients treated at Amsterdam UMC were included, making this a single-center study. Inclusion criteria were patients aged 18 years or younger, the availability of a CT scan of the initial trauma admission, and the presence of a pelvic ring fracture on the CT scan. Patients with only avulsion fractures, isolated iliac wing fractures, or coccygeal fractures were excluded from this study.

### Data Collection

Additional parameters that were collected included age, sex, mechanism of injury, Injury Severity Score (ISS), compound fractures, presence of concomitant acetabular fractures, skeletal maturation using the status of the triradiate cartilage, and presence of pelvic circumferential compression devices.

Pelvic fracture patterns were assessed on CT scans from the initial trauma screening. The descriptions and interpretations were done by 3 observers (A.M., R.H., and D.v.E.): 1 PhD student specializing in clinical research on pelvic fractures, 1 musculoskeletal radiologist, and 1 trauma surgeon experienced in pelvic fracture surgery. These experts reached consensus on the classification and interpretation of the fracture patterns in a structured and systematic discussion using a core list of radiologic criteria and parameters.

### Definitions

The pelvic fracture patterns were described and classified using 4 different approaches. Descriptive classification involved detailing the anatomic sites involved in each fracture pattern. The Tile classification primarily categorizes pelvic fractures based on lateral or vertical instability.^12^ The Young and Burgess (Y&B) classification categorizes pelvic fracture patterns based on the direction of the forces that caused the injury.^13^ Lastly, the Torode and Zieg (T&Z) classification was used to indicate the likelihood of associated injuries and the expected outcome (eg, survival) of the injured child.^14^ Given the lack of consensus regarding when a fracture is considered rotational or vertically unstable in children and adolescents, only the Young and Burgess classification was used to determine whether a fracture pattern could be classified using existing systems.

SI-joint width and pubic symphysis width were measured by a musculoskeletal radiologist. We compared our subjective interpretation of possible SI-joint widening of pubic symphysis diastasis with the age-based normative values from Oetgen.^15^ Further details on whether the SI-joint width was abnormal conform literature or per our expert opinion are provided as Supplemental Digital Content 1, http://links.lww.com/BPO/A965.

Skeletal maturity was determined by evaluating triradiate cartilage status; an immature pelvis was defined as having an open triradiate cartilage, while a mature pelvis was characterized by a closed triradiate cartilage.

Incomplete compression fractures observed in the sacrum or pubic bones were defined as buckle fractures.

Straddle fractures are bilateral fractures of the pubic superior and inferior rami.^16^ In this study, a buckle fracture in a pubic ramus was considered an incomplete fracture and could not be categorized as a straddle fracture.

Avulsion fractures in the SI joint were defined as both avulsion fractures originating from the sacrum and/or originating from the ilium.

For a more comprehensive understanding of the data, the fracture patterns were categorized into unilateral and bilateral sacral or iliac fractures, with avulsion fractures in the SI-joint and/or SI-widening.

Injuries sustained while driving a motor vehicle or as a passenger of a motor vehicle were categorized as “motor vehicle accident (MVA).” If the injury occurred while cycling or walking and involved a collision with a motor vehicle, it was categorized as “bike vs. motorvehicle (MV)” or “pedestrian vs. MV,” respectively. Collectively, these 3 categories constituted “traffic accidents.” Injuries resulting from activities such as horse riding or being hit by a train were classified as “other.”

### Data Analysis

Data were analyzed using IBM SPSS version 28.0. Descriptive statistics were used to describe the patient characteristics, fracture patterns, and classifications. Normality of continuous data was tested with the Kolmogorov-Smirnov test. For parametric data, the mean and SD were reported and for nonparametric data, the median and percentiles. We analyzed the prevalence of different fracture types within our cohort and evaluated the concordance and discordance between the existing classification systems when applied to pediatric pelvic fractures.

### Ethical Review Statement

This study was reviewed by the Medical Ethics Review Committee (METC) of Amsterdam UMC, the Netherlands, which determined that the study does not fall under the scope of the Medical Research Involving Human Subjects Act (WMO) and is therefore classified as non-WMO research.

## RESULTS

Of the 80 pediatric patients with a pelvic ring fracture who were presented to our hospital, 68 patients were included in this study. The median age was 15.5 years (IQR 13 to 17, range 4 to 18). A slight majority of the patients were female (n=36, 53%). Traffic accidents were the main mechanism of injury (n=44, 65%); 18 motor vehicle accidents (27%), 13 pedestrian vs. MV (19%), and 13 bike vs. MV (19%). Furthermore, 21% of the injuries were caused by a fall from height (n=14), 12% by crush injury (n=8), and 3% by other mechanisms of injury (n=2). The median Injury Severity Score (ISS) was 12 (IQR 9 to 19, range 4 to 48). Concomitant acetabular fractures were seen in 12 (18%) patients, and 6 patients (9%) had an open pelvic fracture. A pelvic circumferential compression device was in place at the time of CT imaging in 27 patients (40%).

Fracture patterns with bilateral posterior fractures (n=45, 66%) were more common than unilateral (n=23, 34%). The combination of sacral and/or iliac fractures in combination with SI-widening in the same hemipelvis was seen in 28 patients (41%). The combination of avulsion fractures in the SI joint and SI-joint widening was seen in 9 patients (13%). Anterior fracture patterns were categorized as unilateral or bilateral, with or without pubic symphysis diastasis or symphysiolysis. Bilateral anterior fractures were seen in 52% of the patients (n=35), and unilateral in 32% (n=22). In addition, there were 6 patients (9%) with unilateral fractures and pubic symphysis diastasis or symphysiolysis. Two patients (3%) had symphysiolysis without fractures, and 3 patients (4%) had no anterior injuries. Supplemental Digital content 2, http://links.lww.com/BPO/A966 provides an extensive description of the anatomic fracture sites for each patient’s pelvic fracture patterns.

Most patients had a mature pelvis, and a closed triradiate cartilage was seen in 65% of the patients (n=44). Tile type B1 was seen in 10% of the patients (n=7), type B2 in 53% (n=36), type B3 in 24% (n=16), type C1 in 9% (n=6), type C2 in 1% (n=1) and type C3 in 3% (n=2). Young and Burgess classification type APC2 was seen in 10% (n=7), type LC1 in 22% (n=15)%, type LC2 in 22% (n=15), type LC3 (n=22, 32%), type VS in 12% (n=8), and 1 patient could not be classified (2%). All patients had a T&Z type 4 (n=68, 100%).

**FIGURE 1:**
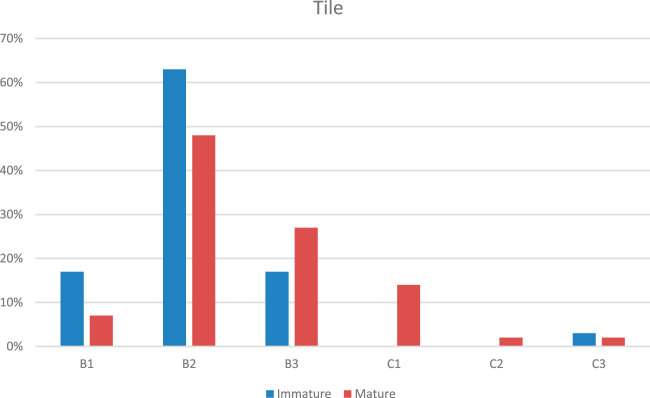
The bar chart illustrates the percentage distribution of fracture patterns according to the Tile classification, categorized by skeletal maturity (immature vs. mature). The classifications are denoted as B1, B2, B3, C1, C2, and C3. Each category shows a comparison between the proportions of fractures in immature and mature skeletal structures. Notably, B2 fractures are the most common in immature skeletons, while mature skeletons show a relatively higher distribution in B2 and B3 fracture types. The chart highlights differences in fracture pattern prevalence between immature and mature skeletal structures.

**FIGURE 2:**
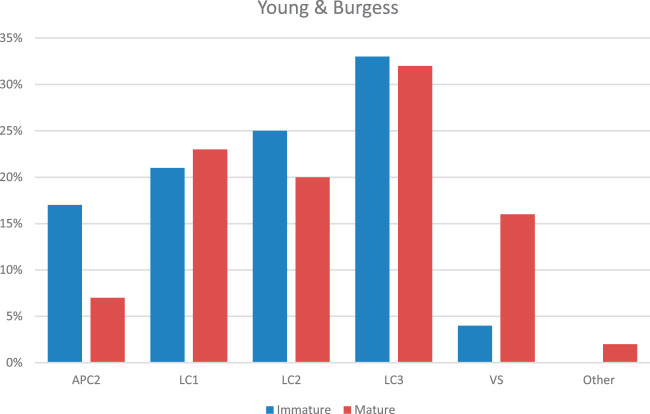
The bar chart depicts the percentage distribution of fracture patterns according to the Young and Burgess classification, categorized by skeletal maturity (immature vs. mature). The classifications are represented as APC2, LC1, LC2, LC3, VS, and other. The chart compares the prevalence of these fracture patterns between immature and mature skeletal structures. LC3 fractures are most prevalent in both immature and mature skeletons, with a higher proportion seen in the immature group. Notable differences are also observed in the distribution of VS fractures, which are more common in mature skeletons. This chart highlights variations in fracture patterns between the 2 groups based on skeletal maturity.

Figures 1 and 2 show the differences in frequency of the Tile and Young and Burgess fracture patterns comparing patients with a mature pelvis to patients with an immature pelvis. VS fractures are more common in patients with an immature pelvis compared with patients with a mature pelvis (16% vs. 4%), and APC2 fractures are more frequently seen in patients with a mature pelvis (17% vs. 7%).

The majority of the patients (n=50, 74%) had fracture patterns that could not be classified according to the classic Young & Burgess classification system. Not fitting into the classic classification system was a problem in both patient groups with a mature pelvis and an immature pelvis; 73% (n=32) of the patients with a mature pelvis and 75% (n=18) of the patients with an immature pelvis did not fit into the classic Young & Burgess classification.

The frequency of specific pediatric pelvic fracture characteristics was also analyzed (Table 1). Avulsion fractures in the SI joint were seen in 21% of the patients (n=14). The majority of the patients with avulsion fractures in the SI joint had a mature pelvis (n=10, 71%). Buckle fractures in the sacrum were seen in 16% (n=11) of the patients, and in the pubic rami in 18% (n=12) of the patients. Fifty-eight percent (n=7) of the patients with a buckle fracture of the sacrum, and 58% (n=6) of the patients with a buckle fracture of the pubic rami had an immature pelvis. Straddle fractures were seen in 25% (n=17) of all patients. The majority of the patients with a straddle fracture had a mature pelvis (n=12, 71%).

**TABLE 1:** Distribution of Specific Pediatric Pelvic Fracture Types Among Skeletally Mature and Immature Patients

Fracture type	Total (n=68)	Mature pelvis (n=44), n (%)	Immature pelvis (n=24), n (%)
Avulsion fractures (SI joint)	14	10 (23)	4 (17)
Buckle fractures (sacrum)	11	5 (11)	6 (25)
Buckle fractures (pubic rami)	12	5 (11)	7 (29)
Straddle fractures	17	12 (27)	5 (21)
Classic pattern	18	12 (27)	6 (25)

Percentages are calculated relative to the total number within each skeletal maturity group.

## DISCUSSION

The results of this study show, using detailed descriptions of the fracture patterns, that pediatric pelvic fractures exhibit unique patterns, potentially due to the specific characteristics of children’s bones. As a result, many pediatric pelvic fractures do not fit adequately within existing classification systems.

It has previously been determined that the pediatric pelvis has different biomechanical characteristics than the adult pelvis, which allows for distinct pediatric fracture patterns and characteristics. A greater portion of haversian canals in the cortex makes young bone more porous and more flexible, allowing for fractures to occur not only by tension but by compression as well.^17^ Previous biomechanical studies have shown that extreme forces are necessary before a fracture will occur in the pediatric pelvis. While in the adult population, forces as low as 2000 N can cause a fracture in the adult pelvis, in children up to 14 years old, forces of 6000 N were needed to cause a fracture.^8^ In 1-year-old children, only traumatic bowing and no fracture occurred in forces up to 10,000 N.^8^ A fracture in the pediatric pelvis should therefore be considered as a signal of extremely high-energy trauma, and the patient should be examined using a comprehensive clinical and radiologic assessment of the patient, with attention to injury severity, potential instability, and associated injuries.

A challenge in classifying pediatric pelvic fracture patterns is accurately interpreting the width of the SI joint and pubic symphysis. These normative values are age-based and sex-based, which makes them difficult to use in a clinical setting or take them into consideration in a classification system. In this study, we noticed that, comparing the values provided in literature to our visual judgment, we did not once agree when both SI joints were measured wide, following normative values. A clear asymmetry between both SI-joint widths determined by visual assessment seemed to be more indicative of SI-joint widening than measuring alone. The influence of a pelvic circumferential binding device, which was used in 40% of the patients in this study, on SI-joint and pubic symphysis width should also be taken into consideration. If positioned correctly, these devices can attain complete reduction of the SI-joint and pubic symphysis displacement,^18^ which makes interpreting possible SI-joint widening challenging and can lead to an underestimation of the fracture pattern. In our study, 24% (n=16) of the patients had a “pediatric LC3” (ped-LC3) fracture pattern without contralateral injury, and half of these patients (n=8, 50%) had a pelvic circumferential binder in place. One should, despite the above-described theory, take the influence of a pelvic binder on possible missed SI-joint widening in the contralateral hemipelvis into account. Manipulation under anesthesia (MUA) could play a role in diagnosing missed SI-joint injuries.

Also, APC2-like fracture patterns in children and adolescents seemed to differ from the adult population. We noticed a high incidence of SI-joint avulsion fractures in children and adolescents with APC2-like fracture patterns, so purely ligamentous APC-fracture patterns are, in our experience, very rare. Avulsion fractures occur due to an imbalance between ligamentous strength and the weaker unfused apophysis. Although younger children have softer bone, avulsion fractures are in general more common in the adolescent population, possibly due to hormonal changes in the growth plates at the time of pubescence.^19^ Our results reflected this as well; 71% of the patients with avulsion fractures in the SI joint had a mature pelvis.

Straddle fractures are not unique to the pediatric pelvis; however, they have a notably higher incidence in this group (25% in our study) compared with adults (11%).^20^ This difference may be due to biomechanical differences in the pediatric pelvis or could be attributed to the high-energy trauma that is required to cause pelvic fractures in children and adolescents. It is important to recognize that straddle fractures are associated with a high incidence of severe urogenital injuries (39%).^20^ Therefore, when straddle fractures are identified on initial imaging, clinicians should be alert to the potential of such complications in the acute setting.

The influence of skeletal maturity on pelvic fracture patterns in children has been discussed in recent literature.^10,11,21^ The status of the triradiate cartilage has been preferred as a sign of skeletal maturity over the Risser stage in these articles. Most articles advocate for a classification and management strategy of pediatric pelvic fractures based on skeletal maturity. In our study, the ped-LC3 fracture pattern was seen more frequently in patients with an immature pelvis (33% vs. 18%); however, nonclassic fracture patterns were observed equally in patients with open (75%) and closed triradiate cartilage (73%). An explanation for this finding could be that the distinction between the mature and immature pelvis should not be based solely on the closure of triradiate cartilage, but should take the peak bone mass into consideration. Although the bone mass increases significantly during the first 2 decades of life, peak bone mass is achieved as late as the end of the second or start of the third decade of life.^22^ The relationship between peak bone mass and fracture risk in the elderly has been extensively researched. However, the impact of this relationship on the distribution of forces and the biomechanics of the pelvis in growing children remains unknown and warrants further investigation. Until further research on this topic has been done, dividing pediatric patients into 2 groups based on skeletal maturity will only help to give an indication of the expected severity and type of associated injuries, but will not be very helpful when deciding if surgical stabilization is needed.

### The Pediatric LC3

**FIGURE 3:**
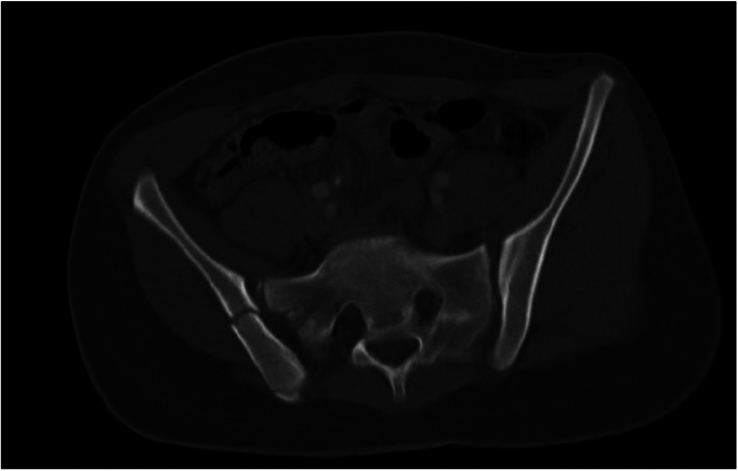
This CT scan shows a pediatric LC3 (lateral compression type 3) fracture pattern in the right hemipelvis. The image highlights a sacral fracture in zone 2, accompanied by a vertical iliac fracture and widening of the sacroiliac (SI) joint. This combination of injuries is characteristic of a pediatric LC3 fracture, indicating significant lateral compression trauma in a pediatric patient.

**FIGURE 4:**
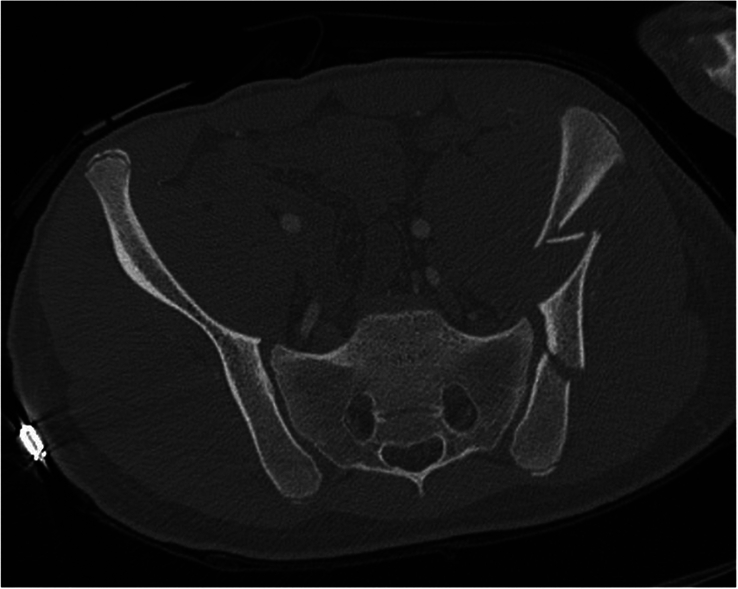
This CT scan illustrates a pediatric LC3 (lateral compression type 3) fracture pattern in the left hemipelvis. The image reveals significant widening of the sacroiliac (SI) joint, along with multiple fractures of the iliac crest arranged in a zig-zag pattern. These features are indicative of a pediatric LC3 injury, which is commonly associated with severe lateral compression trauma in pediatric patients.

**FIGURE 5:**
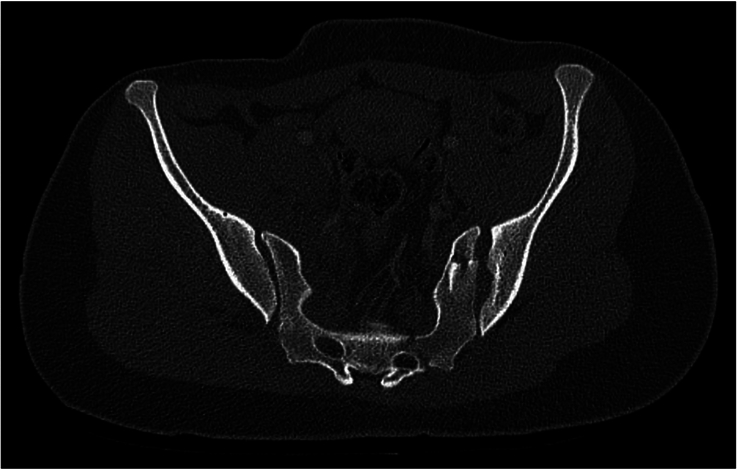
This CT scan demonstrates a pediatric LC3 (lateral compression type 3) fracture pattern in the left hemipelvis. The image shows a zone 2 sacral fracture, an iliac avulsion fracture, and significant widening of the sacroiliac (SI) joint. These features are indicative of a severe lateral compression injury, characteristic of an pediatric LC3 fracture pattern in a pediatric patient.

In this study, we identified a type of pediatric fracture pattern that has not previously been described, the “pediatric-LC3.” The “pediatric LC3”-fracture (ped-LC3) is a combination of pelvic injuries that are not represented in the current adult classification of Young and Burgess,^13^ but were frequently seen during the analysis or the CT scans. We defined the ped-LC3 fracture pattern as a fracture pattern with sacral and/or iliac fractures in combination with widening of the SI joint in that same hemipelvis. The contralateral posterior hemipelvis generally does not show any injuries, in contrast with the adult LC3 fracture pattern. One could explain this phenomenon due to the ligamentous strength and deforming properties described in children, in combination with the high-impact trauma. The pediatric-LC3 (ped-LC3) fracture pattern was seen in 24% (n=16) of the patients; 50% (n=8) had an immature pelvis and 50% (n=8) a mature pelvis. Overall, 33% (n=8) of the patients with an immature pelvis had a ped-LC3 fracture pattern, and 18% (n=8) of the patients with a mature pelvis. Figure 3 shows an example of a ped-LC3 fracture pattern consisting of a sacral fracture (zone 2), vertical iliac fracture and SI-joint widening, all in the right hemipelvis. Figure 4 shows an example of a ped-LC3 fracture pattern consisting of SI-joint widening and multiple iliac crest fractures in a zig-zag pattern in the left hemipelvis. Figure 5 shows an example of a ped-LC3 fracture pattern consisting of a zone 2 sacral fracture, iliac avulsion fracture, and SI-joint widening in the left hemipelvis. A more detailed analysis of the ped-LC3 variants, along with a schematic classification, will be included in a separate follow-up manuscript about pediatric pelvic ring fracture patterns currently in preparation.

### Strengths and Limitations

This study provides a detailed description and extensive classification of pelvic fracture patterns in the pediatric pelvis, contributing to a deeper understanding of these injuries. This comprehensive approach allows for a better characterization and differentiation of fracture types. Furthermore, by identifying and describing previously undescribed pediatric fracture patterns, this study contributes to the current knowledge regarding pediatric pelvic fractures. This might help to improve classification and treatment strategies.

This study involved fracture pattern descriptions and classifications of the Tile, Young and Burgess, and Torode and Zieg classifications. Although standardized evaluation criteria were formulated before the start of the study and consensus was achieved on all cases, the intraobserver and interobserver reliability of these classifications may have introduced bias in the assessment process. This could have affected the consistency and reliability of the reported fracture pattern classifications.

A key limitation of this study is the absence of clinical data, including intraoperative assessments, which restricts our ability to comment definitively on the mechanical stability of the fractures. Since fracture stability was inferred solely from imaging and existing classification systems, we cannot confirm which injuries ultimately required or underwent surgical stabilization. This is a significant limitation, and future studies should incorporate operative findings and clinical outcomes to better assess fracture stability and treatment implications.

## CONCLUSIONS

This study provides more insight into the different fracture patterns of pediatric patients with a pelvic fracture. A large portion of patients with open and closed triradiate cartilage cannot be classified according currently existing classification systems (Young and Burgess). In contrast to the adult APC2 fracture patterns, APC2-like fracture patterns in children and adolescents had a high incidence of SI-joint avulsion fractures, and purely ligamentous APC-fracture patterns were very rare. In addition, the results reveal a previously undescribed fracture pattern, best described as a “pediatric-LC3,” which is most frequently seen in patients with an immature pelvis. Taking the forces necessary to cause a fracture in the pediatric pelvis into consideration, treating physicians should consider a pediatric pelvic fracture a sign of extremely high-energy trauma and should treat the patient accordingly. Future research is necessary to determine the influence of peak bone mass and skeletal maturity on specific pediatric pelvic fractures.

## Supplementary Material

SUPPLEMENTARY MATERIAL
