# Supplementary material for: Assessing Pediatric Pelvic Fracture Patterns: New Insights and Unique Fracture Characteristics
Source: J Pediatr Orthop. 2025 Sep 22;46(2):95–101. doi: 10.1097/BPO.0000000000003107 (PMC12783359; doi:10.1097/BPO.0000000000003107)
Supplement: SUPPLEMENTARY MATERIAL [file bpo-46-0095-s001.docx]

**Supplemental digital content 2: description of the anatomical fracture sites of the pelvic fracture patterns per patient**

|  | Descriptive Fx pattern |
| --- | --- |
| 1 | Sacral Fx (zone 2) and iliac avulsion Fx and SI-joint widening on the left side  bilateral ramus superior and inferior Fx |
| 2 | Sacral Fx (zone 2) on the right side, SI-joint widening on the left side  bilateral ramus superior Fx, bilateral ramus inferior buckle Fx with pubic symphysis diastasis |
| 3 | Iliac avulsion Fx and SI-joint widening on the right side  Pubic symphysis diastasis with apophyseal avulsion fracture |
| 4 | Sacral Fx (zone 1) and SI-joint widening on the right side  bilateral ramus superior and inferior Fx with pubic symphysis diastasis |
| 5 | U-shape sacral Fx and iliac avulsion Fx and SI-joint widening on the left side |
| 6 | Comminuted sacral Fx (zone 2) on the right side, sacral avulsion Fx on the left side, bilateral iliac Fx  bilateral ramus superior and inferior Fx |
| 7 | Comminuted Sacral Fx (zone 2) and iliac crest Fx and Si-joint widening on the left side  ramus inferior Fx on the right side |
| 8 | Sacral buckle Fx (zone 1) and SI-joint widening on the left side  bilateral buckle Fx in ramus superior, buckel FX in ramus inferior on the left side, ramus Fx on the right side |
| 9 | Sacral Fx (zone 1) on the left side, SI-joint widening on the right side  bilateral ramus superior Fx, bilateral buckle Fx in the ramus inferior |
| 10 | SI-joint widening on the left side  ramus superior and inferior Fx on the right side with pubic symphysis diastasis |
| 11 | Comminuted sacral Fx (zone 2) on the left side  bilateral ramus superior and inferior Fx |
| 12 | Iliac crest fracture on the left side  bilateral ramus superior Fx, ramus inferior Fx on the right side, buckle Fx ramus inferior on the left side |
| 13 | Iliac Fx and SI-joint widening on the right side  ramus superior and inferior Fx on the right side |
| 14 | Sacral Fx (zone 1) and iliac crest Fx and SI-joint widening on the left side  bilateral ramus superior Fx, ramus inferior Fx on the right side, buckle Fx ramus inferior on the left side |
| 15 | Sacral Fx (zone 2) and iliac avulsion Fx on the right side, SI-joint widening on the left side  ramus superior and inferior Fx on the right side, buckel Fx ramus superior on the left side |
| 16 | Sacral Fx (zone 2) and SI-joint widening on the left side  bilateral ramus superior and inferior Fx |
| 17 | Sacral Fx (zone 2) and iliac avulsion Fx and SI-joint widening on the left side  bilateral ramus superior and inferior Fx |
| 18 | Sacral Fx (zone 1) on the right side  ramus superior and inferior Fx on the right side |
| 19 | U-shape sacral Fx  multiple ramus superior Fx on the right side |
| 20 | Iliac avulsion Fx and SI-joint widening on the right side  ramus superior and inferior Fx on the left side |
| 21 | Bilateral SI-joint widening  ramus superior and inferior Fx on the left side with pubic symphysis diastasis |
| 22 | Sacral avulsion Fx on the left side  ramus inferior Fx on the left side |
| 23 | Sacral Fx (zone 1) with iliac avulsion Fx on the right side  bilateral ramus superior Fx, ramus inferior Fx on the left side, buckle Fx ramus inferior on the right side |
| 24 | Sacral avulsion Fx (zone 1) and vertical iliac Fx and SI-joint widening on the left side  bilateral buckle Fx ramus superior, buckle Fx ramus inferior on left side |
| 25 | Sacral buckle Fx (zone 1) on the right side  ramus superior Fx on the left side and bilateral ramus inferior Fx |
| 26 | Sacral Fx (zone 2) on the left side  bilateral ramus superior and inferior Fx |
| 27 | U-shape sacral Fx with SI-joint widening on the left side  bilateral ramus superior Fx, ramus inferior Fx on the left side |
| 28 | Sacral Fx (zone 1) on the left side, iliac avulsion Fx on the right side  ramus superior Fx on the right side |
| 29 | Sacral Fx (zone 2) and SI-joint widening on the left side  bilateral ramus superior and inferior Fx with symphysiolysis |
| 30 | Sacral Fx (zone 1) and SI-joint widening on the left side, bilateral iliac crest Fx  bilateral ramus superior and inferior Fx |
| 31 | Sacral Fx (zone 2) on the right side  ramus superior and inferior Fx on the right side |
| 32 | Sacral Fx (zone 1) and vertical iliac Fx and SI-joint widening on the right side  bilateral ramus superior Fx and ramus inferior Fx on the right side |
| 33 | Bilateral sacral buckle Fx (zone 1)  ramus superior Fx on the right side and bilateral ramus inferior Fx |
| 34 | Sacral Fx (zone 2) and vertical iliac Fx on the left side  bilateral ramus superior Fx and ramus inferior Fx on the left side with pubic symphysis diastasis |
| 35 | SI-joint widening on the left side  ramus superior and inferior Fx on the left side with symphysiolysis |
| 36 | Sarcal buckle Fx (zone 1) on the left side  ramus superior and ramus inferior Fx on the left side |
| 37 | Sarcal buckle Fx (zone 1) on the right side  ramus superior and ramus inferior Fx on the right side |
| 38 | Sacral Fx (zone 2) on the right side  ramus superior and ramus inferior Fx on the right side |
| 39 | Sarcal buckle Fx (zone 1) on the right side, iliac wing Fx and SI-joint widening on the left side  ramus superior Fx on the left side |
| 40 | U-shape sacral Fx  ramus inferior Fx on the right side with symphysiolysis |
| 41 | Sacral Fx (zone 2) and vertical iliac Fx and SI-joint widening on the right side  ramus superior and inferior Fx on the right side |
| 42 | Sacral Fx (zone 1) on the left side  ramus superior buckle Fx on the right side, ramus superior Fx on the left side, ramus inferior buckle Fx on the left side |
| 43 | SI-joint widening and multiple vertical iliac Fx on the left side  bilateral ramus superior and ramus inferior Fx |
| 44 | Sacral Fx (zone 2) and iliac avulsion Fx on the left side, SI-joint widening on the right side  ramus superior and ramus inferior Fx on the left side |
| 45 | Sacral buckle Fx (zone 1) on the left, SI-joint widening on the right side  ramus superior Fx on the right side, bilateral ramus inferior Fx |
| 46 | Sacral avulsion Fx (zone 1) on the left side  ramus superior and ramus inferior Fx on the left side |
| 47 | Sacral Fx (zone 2) on the left side  ramus superior and ramus inferior Fx on the left side |
| 48 | U-shape sacral Fx with SI-joint widening on the right side  ramus superior and ramus inferior Fx on the left side with symphysiolysis |
| 49 | Longitudinal sacral Fx (zone 3) with sacral buckle Fx on the right side  ramus superior Fx on the left side, ramus inferior Fx and buckle Fx on the left side, and ramus inferior Fx on the right side |
| 50 | Sacral Fx (zone 1) and SI-joint widening on the left side  bilateral ramus superior Fx and ramus inferior Fx on the right side |
| 51 | Sacral Fx (zone 2) on the right side  ramus superior and ramus inferior Fx on the right side |
| 52 | Sacral avulsion Fx (zone 1) and iliac crest Fx and SI-joint widening on the left side  bilateral ramus superior and ramus inferior Fx with symphysiolysis |
| 53 | Sacral Fx (zone 2) on the left side with SI-joint widening on the right side  ramus superior and ramus inferior Fx on the left side |
| 54 | Sacral buckle Fx (zone 1) and vertical iliac Fx and SI-joint widening on the left side  ramus superior and ramus inferior Fx on the left side |
| 55 | Sacral avulsion Fx (zone 1) on the left side  symphysiolysis |
| 56 | Sacral Fx (zone 1) and iliac avulsion Fx and SI-joint widening on the right side  ramus superior Fx on the left side, ramus inferior Fx on the right side, pubic symphysis diastasis |
| 57 | Sacral Fx (zone 1) and iliac avulsion Fx and SI-joint widening on the left side  bilateral ramus superior Fx, ramus inferior Fx on the left side |
| 58 | Sacral buckle Fx (zone 1) and iliac avulsion Fx and SI-joint widening on the left side  bilateral ramus superior Fx, ramus inferior buckle Fx on the left side |
| 59 | U-shape sacral Fx and horizontal iliac Fx and SI-joint widening on the left side |
| 60 | U-shape sacral Fx and sacral buckle Fx and SI-joint widening on the right side  ramus superior Fx and buckle Fx on the right side, ramus inferior Fx on the right side |
| 61 | SI-joint widening on the left side  pubic symphysis diastasis |
| 62 | U-shape sacral Fx |
| 63 | Sacral Fx (zone 2) on the right side  bilateral ramus superior Fx, ramus inferior Fx on the right side |
| 64 | Iliac wing Fx on the left side  ramus superior and ramus inferior Fx on the left side |
| 65 | Sacral buckle Fx (zone 1) on the left side  ramus superior Fx on the right side and bilateral ramus inferior Fx |
| 66 | Sacral buckle Fx (zone 1) and horizontal iliac Fx on the left side, SI-joint widening on the right side  ramus superior Fx on the right side |
| 67 | U-shape sacral Fx, iliac crest Fx and SI-joint widening on the left side  bilateral ramus superior and ramus inferior Fx |
| 68 | Bilateral sacral Fx (zone 1) and SI-joint widening on the right side  Comminuted ramus superior and ramus inferior Fx on the right side |
